# Association between loneliness and its components and cognitive function among older Chinese adults living in nursing homes: A mediation of depressive symptoms, anxiety symptoms, and sleep disturbances

**DOI:** 10.1186/s12877-022-03661-9

**Published:** 2022-12-13

**Authors:** Qingyan Wang, Chang Zan, Fen Jiang, Yoko Shimpuku, Sanmei Chen

**Affiliations:** 1 Department of Clinical Nursing, School of Nursing, Xuzhou Medical University, 209 Tongshan Road, Xuzhou, 221004 China; 2 Department of Nursing, The Affiliated Hospital of Xuzhou Medical University, 99 West Huaihai Road, Xuzhou, 221006 China; 3 Department of Clinical Nursing, Hunan Normal University School of Medicine, 371 Tongzipo Road, Changsha, 410013 China; 4 Global Health Nursing, Graduate School of Biomedical and Health Sciences, Hiroshima University, 1-2-3 Kasumi, Minami Ward, Hiroshima, 734-8551 Japan

**Keywords:** Loneliness, Cognitive function, Depressive symptoms, Older adults, Nursing home, Chinese

## Abstract

**Objective:**

This study aimed to investigate the associations between loneliness and its components and cognitive function among older Chinese adults living in nursing homes and to test whether depressive symptoms, anxiety symptoms, and sleep disturbances mediate these associations.

**Methods:**

The sample comprised 228 Chinese individuals aged ≥ 65 years living in nursing homes who were free of dementia and psychiatric or serious somatic diseases. Loneliness was evaluated using the UCLA Loneliness Scale. Global cognitive function was assessed using the Beijing version of the Montreal Cognitive Assessment. Multivariable linear regression analyses were performed to examine the associations between loneliness and its components and global cognitive function. A mediation analysis was used to test the potential mediating effects of depressive symptoms, anxiety symptoms, and sleep disturbances.

**Results:**

The mean (SD) age of the participants was 80.8 (6.3) years, and 58.3% were women. Compared with the lowest quartile of loneliness degree, the multivariable-adjusted beta coefficient (95% confidence interval [95% CI]) for the highest quartile was -1.32 (-2.61 to -0.02) (*P* for trend = 0.03). Loneliness components, personal feelings of isolation and the lack of relational connectedness but not the lack of collective connectedness, were also inversely associated with cognitive function. Significant indirect effects on cognitive function were observed for loneliness and its two components (personal feelings of isolation and the lack of relational connectedness) in mediating pathways via depressive symptoms, anxiety symptoms, and sleep disturbances (all *p* < 0.05).

**Conclusions:**

A higher degree of loneliness and its two components, personal feelings of isolation and the lack of relational connectedness, are associated with worse cognitive function among Chinese residents in nursing homes. Depressive symptoms, anxiety symptoms, and sleep disturbances may at least partially mediate these associations.

**Supplementary Information:**

The online version contains supplementary material available at 10.1186/s12877-022-03661-9.

## Introduction

More than 57.4 million people worldwide live with dementia [1]. Dementia is a major cause of disability and dependency among older adults; however, it currently remains incurable [2]. Impairment in cognitive function is a strong risk factor for the development of dementia [3]. Thus, investigations into preventive measures for cognitive impairment are of substantial importance [4]. Loneliness is a subjective and unpleasant experience that occurs when a person’s achieved social relations are less numerous or satisfying than their desires [5–7]. Loneliness has recently attracted increased attention because of its potential role as a modifiable risk factor for cognitive impairment among older adults [8].

Several studies on older adults have examined the association between loneliness and its components and cognitive function; however, most have been conducted among older adults living in the community [9, 10]. To date, few studies have investigated loneliness among older adults living in nursing homes where the environments are distinct from those dwelling in the community. Specifically, older adults in nursing homes live with older adults without kinship and receive services from institution staff instead of families, which can further deepen their loneliness [11, 12]. Risk factors for loneliness have been found to be common among nursing home residents, such as female gender, low income, recent losses of loved ones, disabilities, poor health [12], and psychological conditions of feeling abandoned or shamed because they have no child or resources to ensure non-institutional care [13]. These differences may accumulate and induce loneliness in older adults living in nursing homes to a high degree [14] where their cognitive function may be damaged. However, epidemiological evidence regarding loneliness among nursing home residents and its association with cognitive function remains limited.

Existing evidence on the association between loneliness and cognitive function has yielded mixed results, with some studies showing significant inverse associations between loneliness and its components and cognitive function [8, 15, 16], whereas others suggest no association [17, 18]. In addition, to the best of our knowledge, few studies have investigated the association between the components of loneliness and cognitive function. Exploring the association between the specific components of loneliness helps clarify their discrete effects on cognitive function to develop targeted interventions.

The mechanisms behind the association between loneliness and cognitive function remain yet to be fully understood. It is posited that loneliness can lead to cognitive decline via increased depressive symptomatology and increased anxiety along with decreased sleep quality [19, 20]. High levels of loneliness can threaten feelings of personal worth which can induce depression [5, 22, 23]. Depression alters cognitive function via many paths, such as increasing the deposition of β-amyloid plaques [21]. However, some studies did not support this hypothesis by showing that the association between loneliness and cognitive decline is independent of depression [22]. It remains unclear whether depression mediates the association between loneliness and cognitive function. In addition, loneliness is related to threatened feelings and anxiety [23] and induces augmented stress reactivity and sleep disturbances, all of which, in turn, may lead to cognitive impairment [20, 24–26]. Therefore, we hypothesized that the association between loneliness and cognitive function may be partially attributed to loneliness-associated anxiety symptoms via sleep disturbances.

Thus, the primary purpose of this study was to investigate the association between loneliness and its components and cognitive function in a population of elderly Chinese adults living in nursing homes. The second purpose was to revisit the previously stated hypothesis of the mediating effects of depression on these associations and test our hypothesis that loneliness-associated anxiety symptoms, via sleep disturbances, mediate the association between loneliness and cognitive function.

## Methods

### Study design and participants

Between July 2019 and January 2020, a cross-sectional study using an in-person survey was conducted in 25 nursing homes in Xuzhou, Jiangsu Province, China. This survey comprised a cognitive function test and questionnaire survey conducted by well-trained staff. In this survey, the inclusion criteria were as follows: age 65 years and older, living in nursing homes for no less than six months, and being able to recall events accurately that occurred within one month. The exclusion criteria were as follows: a medical history of physician-diagnosed dementia, psychiatric or serious somatic diseases, or communication impairment. We contacted 237 eligible participants by sending brochures and inviting them to participate. Of these, 230 agreed to participate, with a response rate of 97.0%. After excluding two participants who did not complete the survey, the final sample of this study consisted of 228 participants. The study protocol was approved by the institutional review board of Xuzhou Medical University (approval no. xzhmu-2019015) and was conducted in accordance with the principles of the Declaration of Helsinki. All the participants provided written informed consent.

### Measurement of global cognitive function

Cognitive function was assessed according to the Beijing version of the Montreal Cognitive Assessment (MoCA-Beijing) [27]. The MoCA-Beijing scale has been extensively used to evaluate global cognitive function in China, with high sensitivity and specificity among older populations [27]. This scale includes the following seven cognitive domains with a total score of 30 points: visual-spatial and executive ability, naming, attention and computation, language, abstraction, delayed recall, and orientation. For participants with less than 12 years of education (education level below senior high school) and a total MoCA-Beijing score less than 30, 1 point was added to the total score [28]. Higher MoCA-Beijing scores indicated better cognitive function.

### Measurement of loneliness

Loneliness was assessed using the UCLA Loneliness Scale (version 3), developed by Russell et al. in 1996 [29]. It is a validated and reliable scale designed to measure the subjective feelings of loneliness. The Chinese version has been widely used, with a reported Cronbach’s alpha coefficient of 0.92 [30]. In this study, Cronbach’s alpha coefficient was 0.92. The discriminant validity was good, with a correlation coefficient of -0.51 with the Perceived Social Support Scale [31]. This 20-item scale consists of three components: personal feelings of isolation (11 items, feelings of aloneness, rejection, and withdrawal), the lack of relational connectedness (five items, a feeling of lack of familiarity, closeness, and support), and lack of collective connectedness (four items, a feeling of lack of group identification and cohesion) [7]. Participants were asked to rate each item on a scale from 1 (“I never feel this way”), 2 (“I rarely feel this way”), 3 (“I sometimes feel this way”), to 4 (“I often feel this way”). The total score ranged from 20 to 80 points, with a higher score indicating a greater degree of loneliness.

### Measurement of potential mediators

Depressive symptoms were measured using the Chinese version of the Patient Health Questionnaire (PHQ-9) [32]. The PHQ-9 assesses how often the participant has been bothered by nine depressive symptoms in the past two weeks. The score for each item on the PHQ-9 ranged from 0 to 3 points, with a total score ranging from 0 to 27 points for the nine items. A total score of > 4 points indicated the presence of depressive symptoms [33].

Anxiety symptoms were measured using the Chinese version of the 7-item Generalized Anxiety Disorder Scale (GAD-7) [34]. The GAD-7 mainly asked patients about their mental and emotional changes in the past two weeks. The total score ranged from 0 to 21 points, with a score of > 5 points indicating the presence of anxiety symptoms [35].

Sleep disturbances were measured using the Pittsburgh Sleep Quality Index (PSQI) [36], which assesses sleep quality over the past month. It consists of 19 self-evaluation questions and five other-evaluation questions, with a higher score indicating worse sleep quality. A total PSQI score of < 7 was considered to indicate sleep disturbances [37]

### Measurement of covariates

Data on the following covariates were collected from the questionnaire: demographic and lifestyle factors and health conditions. Demographic factors included age (years), sex (male or female), and education level (elementary or lower, junior high school, senior high school, and college or higher). Current smoking and drinking were defined as yes or no. Physical activity was measured using the International Physical Activity Questionnaire-short form [38], which is a self-report questionnaire asking about the time spent sitting (h/d), whether walking for more than 10 min at least one day in the last week (yes or no) and moderate and vigorous activities in the last seven days (yes or no). Social involvement-related factors included marital status (married and others [widowed, divorced, or never married]) and living status (living alone, living with one roommate, living with two roommates, or living with three roommates).

Body weight and height were measured using light clothing without shoes. Body mass index (BMI) was calculated using body weight and height (kg/m^2^). Being overweight was defined as yes (BMI ≥ 24 kg/m^2^) or no (BMI < 24 kg/m^2^). The medical history of physician-diagnosed diseases, including hypertension, diabetes, hypercholesterolemia, and cerebrovascular disease, was self-reported. Comorbidities were defined according to the medical history of these diseases as no comorbidity, one comorbidity, two comorbidities, or three comorbidities. Hearing impairment was measured using the Hearing Handicap Inventory for the Elderly Screening Questionnaire (with a score of > 8 points indicating hearing impairment) [39].

### Statistical analysis

Residuals of loneliness and its components were checked for normality using visual inspection of normal quantile–quantile (Q-Q) plot. Because the distribution of loneliness and its components were left-skewed, we presented the level of loneliness and its components as medians (interquartile range [IQR]). We categorized the total loneliness scores into quartiles. We also divided the participants into two groups according to the median score for each component of loneliness (low or high).

The characteristics of the participants were summarized according to quartiles of the total loneliness scores and low or high groups of component scores. Categorical and continuous variables are presented as percentages and means (standard deviation [SD]), respectively. Comparisons of characteristics among the quartiles of the total loneliness scores and low or high groups of each component were performed using one-way analysis of variance or t-test for continuous variables, where appropriate, and chi-square test for dichotomous variables.

To investigate the association between loneliness and its components and cognitive function, we built four linear regression models of cognitive function on the quartiles of loneliness (Q2, Q3, or Q4 [reference Q1]) and the low or high groups of each component (high [reference low]), with adjustments for covariates. First, we adjusted for age (continuous, years) and sex (male or female). In the multivariable-adjusted Model 1, we further adjusted for the following covariates: junior middle school, high school, or college or higher (reference elementary or lower), current smoking (yes or no), current drinking (yes or no), sitting time (continuous, hours/day), walking for more than 10 min at least one day in the last week (yes or no), moderate or vigorous activity (yes or no), marital status (married, or widowed/divorced/never married), number of roommates (living alone, one, two, or three), overweight (yes or no), comorbidity (no comorbidities, one, two, or three comorbidities), and hearing impairment (yes or no). In multivariable-adjusted Model 2, we further adjusted for depressive symptoms (yes or no) and the covariates in multivariable-adjusted Model 1. In multivariable-adjusted Model 3, we further adjusted for anxiety symptoms (yes or no) and sleep disturbances (yes or no) plus the covariates in multivariable-adjusted Model 1. The trend association was assessed by assigning the median value to each quartile category of loneliness and entering this variable into the regression models as a continuous variable. The results are presented as unstandardized regression coefficients with 95% CIs and *p*-values. To test the multicollinearity between independent variables, we estimated variance inflation factors (VIFs) for the multivariable linear regression model. We observed no independent variables with a variance inflation factor (VIF) > 2.0 in any multivariable linear regression model.

We further performed a mediation analysis to confirm the potential mediating effect of depressive symptoms (continuous, original score), anxiety symptoms (continuous, original score), and sleep disturbances (continuous, original score) on the association between loneliness and its components and cognitive function using the SPSS PROCESS macro [40]. Based on our hypothesis, the mediation paths were tested according to Hayes guidelines [40] in two mediating paths: the first was that loneliness affects cognitive function via its induced depressive symptoms (including paths α and β; Supplementary Figure 1a), and the second path was via anxiety symptoms and sleep disturbances (including paths γ, ε, and ζ; Supplementary Figure 1b). Since multicollinearity between variables in the mediation analysis muddles the estimates of indirect effects by increasing the width of CIs and their p-values [40], we tested the two paths separately given a VIF value of near 2.0 for depressive symptoms (continuous). The paths were tested with adjustments for the same 15 covariates as Model 1 in linear regression analysis. A 5,000-sample bootstrap procedure was used to estimate β (95% CI) and test the statistical significance of the direct (i.e., the response of cognitive function to changes in loneliness while controlling for mediating variables in the model), indirect (i.e., the response of cognitive function to changes in loneliness through mediators), and total effects (i.e., the response of cognitive function to changes in loneliness in the presence of mediating variables in the model) [41]. The null hypothesis of this test was that the 95% CI of β covered zero [40]. Full mediation is considered to be present when the indirect effect is significant and the β weight of the direct effect of loneliness on cognitive function is attenuated and does not reach the significance level in the presence of mediators in the model, while partial mediation is considered present when the indirect effect is significant and the β weight of the direct effect remains statistically significant [40]. We did the sensitive analysis by excluding the outliers (> 3 SD or < -3 SD) of depressive symptoms, anxiety symptoms, and sleep disturbances in their mediated models.

All statistical analyses were performed using the IBM SPSS 26 software (IBM SPSS Statistics Version 26, SPSS Inc., Chicago, IL, USA). The statistical significance level was set at *p* < 0.05.

## Results

The mean (SD) age of the participants was 80.8 (6.3) years, and 58.3% were women. The median (IQR) of the total score of loneliness was 29 (23 − 39) points, and the median (IQR) of the components was 15.5 (11 − 22) points for the personal feelings of isolation, 7 (5 − 9) points for the lack of relational connectedness and 7 (5 − 8) points for the lack of collective connectedness.

Table 1 shows the characteristics of the participants according to quartiles of the total loneliness scores. Participants who felt lonelier were less likely to walk for more than 10 min at least one day in the last week; more likely to be widowed, divorced, or unmarried; and have depressive symptoms; and anxiety symptoms. The characteristics of the participants according to the low or high groups of loneliness components are shown in Supplementary Table 1.

**Table 1:** Characteristics of participants according to the quartiles of loneliness levels (*n* = 228)

Variables	N	Loneliness levels ^a^				*P-*value ^b^
		Q1(*n* = 67)	Q2(*n* = 46)	Q3 (*n* = 58)	Q4(*n* = 57)	
Age, mean (SD)	228	80.3 (6.2)	81.2 (5.3)	80.8 (6.7)	81.1 (6.9)	0.24
Sex						0.55
Male, %	98	47.8	34.8	41.4	45.6	
Female, %	130	52.2	65.2	58.6	54.4	
Education level						0.58
Elementary or lower, %	99	47.8	50.0	34.5	42.1	
Junior high, %	47	14.9	17.4	27.6	22.8	
Senior high, %	51	26.9	21.7	19.0	21.1	
College or higher, %	31	10.4	10.9	19.0	14.0	
Current smoking						0.66
No, %	198	83.6	87.0	86.2	91.2	
Yes, %	30	16.4	13.0	13.8	8.8	
Current drinking						0.14
No, %	194	86.6	89.1	75.9	89.5	
Yes, %	34	13.4	10.9	24.1	10.5	
Sitting (h/d), mean (SD)	228	7.7 (3.0)	8.3 (3.6)	8.7 (3.4)	8.4 (3.8)	0.21
Walking for more than 10 min at least one day in the last week						**0.02**
No, %	23	4.5	4.3	12.1	19.3	
Yes, %	205	95.5	95.7	87.9	80.7	
Moderate or vigorous activity						0.14
No, %	198	82.1	89.1	82.8	94.7	
Yes, %	30	17.9	10.9	17.2	5.3	
Marital status						**0.02**
Married, %	60	34.3	13.0	34.5	19.3	
Widowed/divorced/never married, %	168	65.7	87.0	65.5	80.7	
Living status						
Living alone	70	23.9	43.5	27.6	31.6	0.11
Living with one roommate	128	68.7	43.5	60.3	47.4	
Living with two roommates	25	6.0	13.0	10.3	15.8	
Living with three roommates	5	1.5	0.0	1.7	5.3	
Overweight						0.66
No, %	85	32.8	39.1	43.1	35.1	
Yes, %	143	67.2	60.9	56.9	64.9	
Comorbidities						0.85
No comorbidity, %	69	29.9	30.4	29.3	31.6	
One comorbidity, %	94	44.8	43.5	43.1	33.3	
Two comorbidities, %	47	16.4	21.7	22.4	22.8	
Three comorbidities, %	18	9.0	4.4	5.2	12.3	
Hearing impairments						0.19
No, %	190	91.0	80.4	82.8	77.2	
Yes, %	38	9.0	19.6	17.2	22.8	
Depressive symptoms						**0.08**
No, %	145	74.6	65.2	60.3	52.6	
Yes, %	83	25.4	34.8	39.7	47.4	
Anxiety symptoms						**0.001**
No, %	204	100.0	93.5	82.8	80.7	
Yes, %	24	0.0	6.5	17.2	19.3	
Sleep disturbances						0.12
No, %	140	70.1	67.4	51.7	56.1	
Yes, %	88	29.9	32.6	48.3	43.9	
Cognitive function	228	21.1 (4.2)	20.1 (4.3)	20.0 (5.2)	18.7 (5.0)	**0.04**

Abbreviations: SD: standard deviation; Q: quartile

^a^ The cutoffs of the quartiles of loneliness were 23, 28, and 39 points

^b^ The one-way analysis of variance test for continuous variables and chi-square test for categorical variables

Table 2 shows the results of the linear regression models of cognitive function for the components of loneliness. A higher degree of loneliness was significantly associated with worse global cognitive function (*P* for trend = 0.03). The multivariable-adjusted β (95% CI) was -1.32 (-2.61 to -0.02) for the highest quartile compared with the lowest quartile, with adjustments for covariates (multivariable-adjusted Model 1). A significant inverse association was observed for the personal feelings of isolation, and a marginally significant inverse association was also seen for the component of the lack of relational connectedness, with the multivariable-adjusted β coefficients (95% CIs) of -1.26 (-2.22 to -0.31) and -0.93 (-1.87 to 0.00) for the high group compared with the low group, respectively. The lack of collective connectedness was not significantly associated with global cognitive function (*p* = 0.25). After further controlling for depressive symptoms, anxiety symptoms, and sleep disturbances, the associations between loneliness and the lack of relational connectedness and cognitive function were attenuated and became insignificant, while the associations for the personal feelings of isolation were attenuated but remained significant (*p* = 0.03 for the model with further adjustment for depressive symptoms) or marginally significant *(p* = 0.053 for the model with further adjustment for anxiety symptoms and sleep disturbances).

**Table 2:** Association between loneliness and cognitive function by multivariable linear regressions models (*n* = 228)

	Age- and sex-adjusted Model		Multivariable-adjusted Model 1^a^		Multivariable-adjusted Model 2 ^b^		Multivariable-adjusted Model 3 ^c^	
	*β* coefficients ^d^ (95% CI)	*P*-value	*β* coefficients ^d^ (95% CI)	*P*-value	*β* coefficients ^d^ (95% CI)	*P*-value	*β* coefficients ^d^ (95% CI)	*P*-value
Loneliness ^e^								
Q1 (low)	Reference		Reference		Reference		Reference	
Q2	-0.73 (-2.46 to 1.00)	0.41	-0.24 (-1.58 to 1.10)	0.72	-0.18 (-1.50 to 1.15)	0.79	-0.21 (-1.54 to 1.12)	0.76
Q3	-0.98 (-2.59 to 0.64)	0.24	-1.41 (-2.69 to -0.14)	**0.03**	-1.24 (-2.50 to 0.03)	0.06	-1.11 (-2.41 to 0.18)	0.09
Q4 (high)	-2.32 (-3.94 to -0.69)	**0.01**	-1.32 (-2.61 to -0.02)	**0.046**	-1.08 (-2.37 to 0.21)	0.10	-1.07 (-2.38 to 0.24)	0.11
Association trend ^f^	-0.09 (-0.15 to -0.03)	**0.01**	-0.05 (-0.10 to -0.01)	**0.03**	-0.04 (-0.09 to 0.00)	0.07	-0.04 (-0.09 to 0.01)	0.09
Personal feelings of isolation ^g^								
Low	Reference		Reference		Reference		Reference	
High	-1.44 (-2.63 to -0.25)	**0.02**	-1.26 (-2.22 to -0.31)	**0.01**	-1.09 (-2.04 to -0.13)	**0.03**	-0.97 (-1.95 to 0.01)	**0.053**
Lack of relational connectedness ^h^								
Low	Reference		Reference		Reference		Reference	
High	-1.48 (-2.68 to -0.29)	**0.02**	-0.93 (-1.87 to 0.00)	**0.051**	-0.72 (-1.66 to 0.22)	0.13	-0.71 (-1.65 to 0.24)	0.14
Lack of collective connectedness ^i^								
Low	Reference		Reference		Reference		Reference	
High	-1.19 (-2.48 to 0.09)	0.07	-0.01 (-1.63 to 0.42)	0.25	-0.57 (-1.58 to 0.44)	0.27	-0.48 (-1.51 to 0.54)	0.35

Abbreviations: CI: confidence interval; Q: quartile

^a^ Adjusted for age, sex (male or female), education level (elementary or lower, junior high, senior high, college or higher), current smoking (yes or no), current drinking (yes or no), sitting (h/d), walking for more than 10 min at least one day in the last week (yes or no), moderate or vigorous activity (yes or no), marital status (married or widowed, divorced, or unmarried), living status (living alone, living with one roommate, living with two roommates, living with three roommates), overweight (yes or no), number of comorbidities, hearing impairments (yes or no)

^b^ Additionally adjusted for covariates included in multivariable-adjusted Model 1 plus the presence of depressive symptoms (yes or no)

^c^ Additionally adjusted for covariates included in multivariable-adjusted Model 1 plus the presence of anxiety symptoms (yes or no) and sleep disturbances (yes or no)

^d^ *β* coefficients are the unstandardized coefficients

^e^ The cutoffs of the score of loneliness were 23, 28, and 39

^f^ Linear trend across quartiles of loneliness levels was tested by entering the median values of each quartile into the multivariable linear regression model

^g^ The cutoff of the score of personal feelings of isolation was 15

^h^ The cutoff of the score of the lack of relational connectedness was 6

^i^ The cutoff of the score of the lack of collective connectedness was 7

**Fig. 1:**
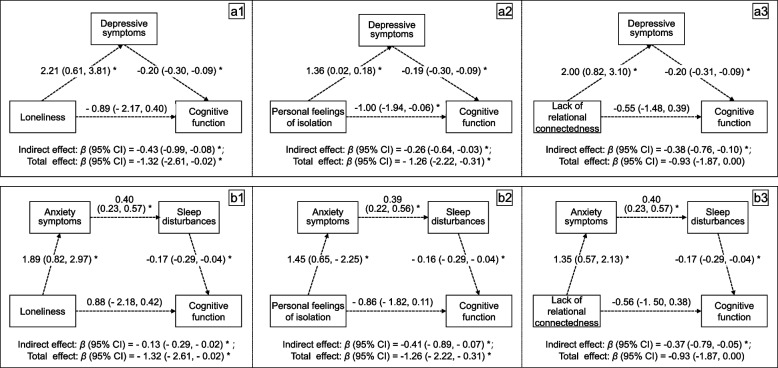
Depressive symptoms (a1 to a3), anxiety symptoms, and sleep disturbances (b1 to b3) mediate the association between loneliness and its two components—personal feelings of isolation and the lack of relational connectedness—and cognitive function. The dotted arrows represent the direct and indirect effects of loneliness (the highest quartile with reference to the lowest quartile) and its two components (the high group with reference to the low group) on cognitive function. The values on the dotted arrows represent the multivariable-adjusted beta coefficients (95% confidence interval [95% CI]) of regression. * *p* < 0.05. The depressive symptoms, anxiety symptoms, and sleep disturbances were continuous variables. All models were adjusted for age, sex (male or female), education level (elementary or lower, junior high, senior high, and college or higher), current smoking (yes or no), current drinking (yes or no), sitting (h/d), walking for more than 10 min at least one day in the last week (yes or no), moderate or vigorous activity (yes or no), marital status (married or widowed, divorced, or unmarried), living status (living alone, living with one roommate, living with two roommates, living with three roommates), overweight (yes or no), number of comorbidities, and hearing impairment (yes or no)

The mediation analyses showed that depressive symptoms, anxiety symptoms, and sleep disturbances fully mediated the association between loneliness and its component of the lack of relational connectedness and cognitive function, while partially mediated the association for the component of the personal feelings of isolation. Figures 1 a1, a2, and a3 show the results of the mediating path via depressive symptoms. There were significant indirect effects of loneliness and the two components, insignificant direct effects of loneliness and the component of the lack of relational connectedness (both *p* > 0.1), but a significant direct effect of the component of personal feelings of isolation (*p* = 0.04). Figure 1 b1, b2, and b3 show the results of the mediating paths via anxiety symptoms and sleep disturbances. There were significant indirect effects of loneliness and the two components, insignificant direct effects of loneliness and the component of the lack of relational connectedness (both *p* > 0.1), but a marginally significant direct effect for the component of personal feelings of isolation (*p* = 0.08). The detailed β coefficients (95% CI) and *p*-values of the total, direct, and indirect effects of both mediating paths are shown in Supplementary Tables 2 and 3. After excluding the outliers of depressive symptoms (*n* = 4), anxiety symptoms (*n* = 8), and sleep disturbances (*n* = 2) in their mediated models in the sensitivity analysis, the results did not materially change. The sensitivity analysis results of the total, direct, and indirect effects of both mediating paths are shown in Supplementary Tables 4 and 5.

## Discussion

In the present study of a population of older Chinese adults living in nursing homes, we found that loneliness and its two components (except for the component of the lack of collective connectedness)—personal feelings of isolation and the lack of relational connectedness—were significantly inversely associated with cognitive function with adjustment for demographic, lifestyle, and health condition factors. Further adjustment for depressive symptoms, anxiety symptoms, and sleep disturbances attenuated the observed associations. Full and partial mediating effects of depressive symptoms, anxiety symptoms, and sleep disturbances were further observed in the mediating pathway analysis.

### Inverse association between loneliness and its components and cognitive function

Our results showing an inverse association between loneliness and cognitive function are consistent with those of previous studies that found loneliness to be a risk factor for cognitive impairment [8, 42]. However, several previous studies have failed to find a significant association between loneliness and cognitive function [9, 18]; this difference may be related to the measurements used. In those studies, cognitive function was measured using the Mini-Mental State Examination [9], which is less sensitive than the MoCA in measuring the subtle cognitive decline in older people without dementia [43, 44], or loneliness was measured using a single item [18], which is prone to measurement error [45]. In the present study, we used the MoCA to measure the total cognitive function, which is sensitive and can capture subtle cognitive decline [43, 44]. Instead of using a single item, we used the 20-item UCLA Loneliness Scale to measure loneliness. Our findings confirmed an inverse association between loneliness and cognitive function in older adults living in nursing homes.

We also found inverse associations between personal feelings of isolation and the lack of relational connectedness with cognitive function. The association between personal feelings of isolation and cognitive function could be explained by the painful feelings of aloneness, rejection, and withdrawal experienced by residents in nursing homes [13], which induce self-doubt and depression, respectively [5, 21, 46, 47] and activate pain-related neural regions [48], resulting in cognitive impairment [49]. The inverse association between the lack of relational connectedness and cognitive function is consistent with findings from previous studies [7, 50]. Older persons tend to select familiar and reliable relationships [51]. However, nursing home residents are far from their familiar friends and relatives and suffer from a painful feeling of lack of relational connectedness [12], which may further damage cognitive function. By contrast, we found no association between the lack of connective connectedness and cognitive function. Our finding was inconsistent with results from prior studies among older adults living in the community which observed a positive association of connective connectedness (such as community support) with cognitive function [52]. This difference might be explained by the different living settings of older adults. Chinese older adults who are living in nursing homes are shown to have few desires or needs for collective connectedness [13]. This is one of their passive perceptive coping method to adapt to their social environment in nursing homes, particularly for older adults who find it difficult to seek a social network in the nursing home as a result of their impaired health or more introverted personality [53]. The Chinese culture towards nursing homes could also explain the lack of an association between connective connectedness and cognitive function. Living in a nursing home is always the last and worst choice in Chinese filial piety culture [13]. The residents’ shame because of living in nursing homes may further weaken their needs or desires for collective connectedness in nursing homes [54]. Consequently, a lack of collective connectedness may not activate cognitive-damaging mechanisms.

### Mediating paths of depressive symptoms, anxiety symptoms, and sleeping disturbances

In the present study, we observed significant mediating effects on the association between loneliness and its two components on cognitive function via depressive symptoms. Loneliness can lead to negative feelings of betrayal, abandonment, and self-doubt that threaten feelings of personal worth and undermine confidence in the ability to develop and maintain interpersonal relationships, which can induce depression as a result [5, 46, 47]. In addition, we found that participants in the highest quartile of loneliness were more likely to have depressive symptoms than those in the lowest quartile (47% vs. 25%). Depression alters glucocorticoid steroids and hippocampal atrophy, increases the deposition of β-amyloid plaques, changes inflammation, and decreases nerve growth factors [21]. Thus, it is biologically feasible that depression mediates the association between loneliness and cognitive function.

Furthermore, we observed significant mediating effects on the association between loneliness and its two components on cognitive function via anxiety symptoms and sleep disturbances. Loneliness, also called perceived social isolation, leads people to feel more anxiety in social situations [55]. This threatened feeling may induce augmented stress reactivity, which is associated with prolonged activation of the hypothalamic–pituitary–adrenal axis and the sympathoadrenal system [20]. This disrupted brain response may further lead to sleep disturbances [20], which damage cognitive function through weakened brain plasticity, disturbed activity-dependent down-selection of synapses, and decreased global brain activity [25, 26]. The results of this study support the mediating effect of anxiety symptoms and sleep disturbances on the association between loneliness and cognitive impairment.

We observed a significant direct effect of the component of personal feelings of isolation on cognitive function, but not of loneliness and the component of a lack of relational connectedness. One possible explanation is that the mediators may be a surrogate of loneliness and the component of a lack of relational connectedness, but not for the component of personal feelings of isolation, suggesting the importance of targeting mediators together with specific loneliness components to prevent cognitive decline. Of note, we adjusted for many covariates that may potentially mediate or moderate the associations between loneliness and cognitive function [56–58], which could have greatly attenuated the direct effects of loneliness and the component of the lack of relational connectedness. Nevertheless, we cannot rule out the possibility that the insignificant direct effect could be due to the limited statistical power resulting from the small sample size. The significant direct effect of the component of the personal feelings of isolation on cognitive function suggests the importance of exploring other potential mechanisms linking them, such as the overlapping neuroanatomical substrates of cognition and loneliness [59] and the loneliness-activated pain damaging cognitive function [48, 49].

Our findings imply that interventions targeting decreasing specific loneliness components ꟷ the personal feelings of isolation and lack of relational connectedness ꟷ may be beneficial for preventing or slowing down cognitive decline among older adults living in the nursing home, such as laughter therapy [60] and video chatting with family members [61]. Interventions that can decrease loneliness together with depression, anxiety, and sleep disturbance simultaneously (i.e. horticultural therapy, etc.[60, 62, 63]) could be beneficial as well.

The strengths of this study include its use of the measurement of loneliness, which provided a complete assessment of the degree of loneliness and enabled us to explore loneliness components in association with cognitive function. The present study measured varying potential confounding factors across demographic, lifestyle, and health factors, as compared with previous studies on the association between loneliness and cognitive function. This study was one of the few to test the mediated pathways of the association between loneliness and cognitive function via depressive symptoms, anxiety symptoms, and sleep disturbances. This study has several limitations. First, the sample size of the present study was relatively small, which may have contributed to the instability of the 95% CIs of the regression coefficient estimates and can account for the borderline significance of the association for the loneliness component of the lack of relational connectedness. Second, the cross-sectional design of the present study limited inferences of causality between loneliness and cognitive function. Finally, the participants were recruited from one city in China. We urge caution in generalizing our findings to populations with different backgrounds.

## Conclusion

In conclusion, the present study of a population of nursing home residents in China demonstrated that loneliness and its two components—personal feelings of isolation and the lack of relational connectedness—were significantly inversely associated with cognitive function and that depressive symptoms, anxiety symptoms and sleep disturbances may at least partially mediate the associations between loneliness and its components and cognitive function. Our findings encourage further intervention studies to target reducing specific loneliness components and integrate the mediating factors into strategies for reducing loneliness to prevent or slow the decline in cognitive function.

## Supplementary Information

**Additional file 1: Supplementary file1.**

## Data Availability

There are ethical or legal restrictions on sharing the datasets used for the current study for containing potentially identifying patients’ information; and the dataset can be accessed from the ethics committee of Xuzhou Medical University (Phone: + 86–0516-85,748,426) or from the corresponding author upon reasonable request (Email: chens@hiroshima-u.ac.jp).

## Abbreviations

SD: Standard deviation
CI: Confidence interval
MoCA-Beijing: Beijing version of Montreal Cognitive Assessment
PHQ-9: Patient Health Questionnaire-9
GAD-7: Generalized Anxiety Disorder Scale-7
PSQI: Pittsburgh Sleep Quality Index
BMI: Body mass index
Q-Q: Quantile-quantile
IQR: Interquartile range
Q: Quartile
VIF: Variance inflation factor
