# Supplementary material for: Association between loneliness and its components and cognitive function among older Chinese adults living in nursing homes: A mediation of depressive symptoms, anxiety symptoms, and sleep disturbances
Source: BMC Geriatr. 2022 Dec 13;22:959. doi: 10.1186/s12877-022-03661-9 (PMC9746079; doi:10.1186/s12877-022-03661-9)
Supplement: Supplementary file 1 — Additional file 1: Supplementary file1. [file 12877_2022_3661_MOESM1_ESM.pdf]

Supplementary Table 1 Characteristics of participants according to the levels of loneliness subscales (n = 228)

Variables	N	Personal feelings of isolation [a]		*P*-value [b]	Lack of relational connectedness [c]		*P*-value [b]	Lack of collective connectedness [d]		*P*-value [b]
		Low (n=114)	High (n=114)		Low (n=112)	High (n=116)		Low (n=155)	High (n=73)	
Age, mean (SD)	228	80.8 (5.5)	80.9 (7.0)	0.93	80.3 (6.2)	81.3 (6.4)	0.21	80.8 (6.3)	81.0 (6.4)	0.81
Sex				0.79			0.62			0.30
Male, %	98	43.9	42.0		44.6	41.4		40.6	47.9	
Female, %	130	56.1	58.0		55.4	58.6		59.4	52.1	
Education level				0.11			0.80			0.89
Elementary or lower, %	99	50.0	36.8		42.9	44.0		45.2	39.7	
Junior high, %	47	17.5	23.7		19.6	21.5		20.0	21.9	
Senior high, %	51	22.8	21.9		25.0	19.8		21.9	23.3	
College or higher, %	31	9.6	17.5		12.5	14.7		12.9	15.1	
Current smoking				0.70			0.62			0.13
No, %	198	86.0	87.7		85.7	87.9		84.5	91.8	
Yes, %	30	14.0	12.3		14.3	12.1		15.5	8.2	
Current drinking				1.00			0.63			0.66
No, %	194	85.1	85.1		83.9	86.2		85.8	83.6	
Yes, %	34	14.9	14.9		16.1	13.8		14.2	16.4	
Sitting (h/d), mean (SD)	228	7.9 (3.4)	8.9 (3.5)	**0.03**	8.3 (3.5)	8.5 (3.4)	0.59	8.0 (3.1)	9.3 (4.0)	**0.01**
Walking for more than 10 min at least one day in the last week				**0.002**			**0.03**			0.09
No, %	23	96.5	83.3		94.6	85.3		92.3	84.9	
Yes, %	205	3.5	16.7		5.4	14.7		7.7	15.1	
Moderate or vigorous activity				0.24			0.38			0.13
No, %	198	84.2	89.5		84.8	88.8		84.5	91.8	
Yes, %	30	15.8	10.5		15.2	11.2		15.5	8.2	
Marital status				0.76			0.29			0.09
Married, %	60	72.8	74.6		70.5	76.7		70.3	80.8	
Widowed/divorced/never	168	27.2	25.4		29.5	23.3		29.7	19.2	

married, %										
Living status				0.25			0.76			0.08
Living alone	70	28.9	32.5		31.3	30.2		29.7	32.9	
Living with one roommate	128	61.4	50.9		58.0	54.3		60.6	46.6	
Living with two roommates	25	8.8	13.2		8.9	12.9		7.7	17.8	
Living with three roommates	5	0.9	3.5		1.8	2.6		1.9	2.7	
Overweight				0.13			0.12			0.60
No, %	85	32.5	42.1		32.1	42.2		36.1	39.7	
Yes, %	143	67.5	57.9		67.9	57.8		63.9	60.3	
Comorbidities				0.25			0.25			0.92
No comorbidity, %	69	30.7	29.8		25.9	34.5		31.6	27.4	
One comorbidity, %	94	46.5	36.0		47.3	35.3		40.0	43.8	
Two comorbidities, %	47	16.7	24.6		20.5	20.7		20.6	20.5	
Three comorbidities, %	18	6.1	9.6		6.3	9.5		7.7	8.2	
Hearing impairments				0.48			**0.04**			0.07
No, %	190	85.1	81.6		88.4	78.4		86.5	76.7	
Yes, %	38	14.9	18.4		11.6	21.6		13.5	23.3	
Depressive symptoms				**0.02**			**0.01**			0.31
No, %	145	71.1	56.1		72.3	55.2		65.8	58.9	
Yes, %	83	28.9	43.9		27.7	44.8		34.2	41.1	
Anxiety symptoms				**<0.001**			**0.001**			**0.01**
No, %	204	97.4	81.6		96.4	82.8		92.9	82.2	
Yes, %	24	2.6	18.4		3.6	17.2		7.1	17.8	
Sleep disturbances				**0.003**			**0.049**			0.60
No, %	140	71.1	51.8		67.9	55.2		62.6	58.9	
Yes, %	88	28.9	48.2		32.1	44.8		37.4	41.1	
Cognitive function	228	20.8 (4.3)	19.3 (5.1)	**0.02**	20.9 (4.2)	19.2 (5.1)	**0.01**	20.4 (4.7)	19.3 (4.8)	0.11

Abbreviations: SD: standard deviation

[a] The score range of personal feelings of isolation was 11 to 44, and the cutoff was15.

[b] Ther t-test for continuous variables and chi-square test for categorical variables.

[c] The score range of the lack of relational connectedness was 5 to 20, and the cutoff was 6.
[d] The score range of the lack of collective connectedness was 4 to 14, and the cutoff was 7.

Supplementary Table 2 Mediation analysis of associations between loneliness and cognitive impairment, mediated by depressive symptoms, anxiety symptoms, and sleep disturbances [a] (n = 228)

Paths	Path via depressive symptoms		Path via anxiety symptoms and sleep disturbances	
	β coefficients [b] (95% CI)	*P*-value	*β* coefficients [b] (95% CI)	*P*-value
Path α or γ (reference Q1)				
Q2	0.68 (-0.98 to 2.34)	0.42	0.44 (-0.67 to 1.56)	0.44
Q3	1.06 (-0.52 to 2.64)	0.19	1.41 (-0.34 to 2.47)	**0.01**
Q4	2.21 (-0.61 to 3.81)	**0.007**	1.89 (-0.82 to 2.97)	**0.001**
Path β	-0.20 (-0.30 to -0.09)	**<0.001**	—	—
Path ε	—	—	0.40 (-0.23 to 0.57)	**<0.001**
Path ζ	—	—	-0.17 (-0.29 to -0.04)	**0.01**
Direct effect (reference Q1)				
Q2	-0.11 (-1.42 to 1.20)	0.87	-0.09 (-1.40 to 1.22)	0.90
Q3	-0.21 (-2.45 to 0.04)	0.06	-1.03 (-2.29 to 0.24)	0.11
Q4	-0.89 (-2.17 to 0.40)	0.18	0.88 (-2.18 to 0.42)	0.18
Indirect effect (reference Q1)				
Q2	-0.13 (-0.49 to 0.15)	Insignificant	-0.03 (-0.12 to 0.02)	Insignificant
Q3	-0.21 (-0.57 to 0.08)	Insignificant	-0.09 (-0.24 to -0.01)	**Significant**
Q4	-0.43 (-0.99 to -0.08)	**Significant**	-0.13 (-0.29 to -0.02)	**Significant**
Total effect (reference Q1)				
Q2	-0.24 (-1.58 to 1.10)	0.72	-0.24 (-1.58 to 1.10)	0.72
Q3	-1.41 (-2.69 to -0.14)	**0.03**	-1.41 (-2.69 to -0.14)	**0.03**
Q4	-1.32 (-2.61 to -0.02)	**0.046**	-1.32 (-2.61 to -0.02)	**0.046**

Abbreviations: CI: confidence interval

[a] The depressive symptoms, anxiety symptoms, and sleep disturbances were continuous variables. All models were adjusted for age, sex (male or female), education level (elementary or lower, junior high, senior high, and college or higher), current smoking (yes or no), current drinking (yes or no), sitting (h/d), walking for more than 10 min at least one day in the last week (yes or no), moderate or vigorous activity (yes or no), marital status (married or widowed, divorced, or unmarried), living status (living alone, living with one roommate, living with two roommates, living with three roommates), overweight (yes or no), number of comorbidities, and hearing impairment (yes or no).

[b] β coefficients are the unstandardized coefficients.

Supplementary Table 3 Mediation analysis models of associations between loneliness components and cognitive function, mediated by depressive symptoms, anxiety symptoms, and sleep disturbances [a] (n = 228)

Path models	Personal feelings of isolation		Lack of relational connectedness	
	β coefficients [b] (95% CI)	*P*-value	β coefficients [b] (95% CI)	*P*-value
Path via depressive symptoms				
Path α (reference Low)	1.36 (-0.02 to 0.18)	**0.02**	2.00 (-0.82 to 3.10)	**0.001**
Path β	-0.19 (-0.30 to -0.09)	**<0.001**	-0.20 (-0.31 to -0.09)	**<0.001**
Direct effect (reference Low)	-1.00 (-1.94 to -0.06)	**0.04**	-0.55 (-1.48 to 0.39)	0.31
Indirect effect (reference Low)	-0.26 (-0.64 to -0.03)	**Significant**	-0.38 (-0.76 to -0.10)	**Significant**
Path via anxious symptoms and sleep disturbances				
Path γ (reference Low)	1.45 (-0.65 to -2.25)	**<0.001**	1.35 (-0.57 to 2.13)	**0.001**
Path ε	0.39 (-0.22 to 0.56)	**<0.001**	0.40 (-0.23 to 0.57)	**<0.001**
Path ζ	-0.16 (-0.29 to -0.04)	**0.01**	-0.17 (-0.29 to -0.04)	**0.01**
Direct effect (reference Low)	-0.86 (-1.82 to 0.11)	**0.08**	-0.56 (-1.50 to 0.38)	0.24
Indirect effect (reference Low)	-0.41 (-0.89 to -0.07)	**Significant**	-0.37 (-0.79 to -0.05)	**Significant**
Total effect (reference Low)	-1.26 (-2.22 to -0.31)	**0.01**	-0.93 (-1.87 to 0.00)	0.051

Abbreviations: CI: confidence interval

[a] The depressive symptoms, anxiety symptoms, and sleep disturbances were continuous variables. All models were adjusted for age, sex (male or female), education level (elementary or lower, junior high, senior high, and college or higher), current smoking (yes or no), current drinking (yes or no), sitting (h/d), walking for more than 10 min at least one day in the last week (yes or no), moderate or vigorous activity (yes or no), marital status (married or widowed, divorced, or unmarried), living status (living alone, living with one roommate, living with two roommates, living with three roommates), overweight (yes or no), number of comorbidities, and hearing impairment (yes or no).

[b] β coefficients are the unstandardized coefficients.

Supplementary Table 4 Sensitive analysis of mediation analysis of associations between loneliness and cognitive impairment, mediated by depressive symptoms, anxiety symptoms, and sleep disturbances [a]

Paths	Path via depressive symptoms (n = 224)		Path via anxiety symptoms and sleep disturbances (n = 218)	
	β coefficients [b] (95% CI)	*P*-value	*β* coefficients [b] (95% CI)	*P*-value
Path α or γ (reference Q1)				
Q2	0.55 (-0.88 to 1.97)	0.45	0.04 (-0.66 to 0.74)	0.92
Q3	1.36 (0.01 to 2.71)	**0.048**	0.74 (0.07 to 1.41)	**0.03**
Q4	1.51 (0.13 to 2.88)	**0.03**	1.16 (0.49 to 1.84)	**0.001**
Path β	-0.16 (-0.28 to -0.04)	**0.01**	—	—
Path ε	—	—	0.50 (0.23 to 0.76)	**<0.001**
Path ζ	—	—	-0.18 (-0.32 to -0.04)	**-0.01**
Direct effect (reference Q1)				
Q2	-0.16 (-1.44 to 1.13)	0.81	-0.16 (-1.50 to 1.17)	0.81
Q3	-1.32 (-2.55 to -0.09)	**0.04**	-1.20 (-2.49 to 0.08)	0.07
Q4	-0.79 (-2.05 to 0.47)	0.22	-0.67 (-1.99 to 0.66)	0.32
Indirect effect (reference Q1)				
Q2	-0.09 (-0.37 to 0.15)	Insignificant	-0.003 (-0.06 to 0.05)	Insignificant
Q3	-0.22 (-0.57 to 0.01)	Insignificant	-0.06 (-0.17 to -0.002)	**Significant**
Q4	-0.24 (-0.63 to -0.001)	**Significant**	-0.10 (-0.25 to -0.01)	**Significant**
Total effect (reference Q1)				
Q2	-0.24 (-1.54 to 1.06)	0.71	-0.14 (-1.49 to 1.21)	0.84
Q3	-1.53 (-2.77 to -0.30)	**0.02**	-1.44 (-2.73 to -0.15)	**0.03**

Q4	-1.03 (-2.29 to 0.23)	**0.11**	-0.99 (-2.30 to 0.32)	0.14

Abbreviations: CI: confidence interval

[a] We did the sensitivity analysis by excluding the outliers (>3 SD or <-3 SD) of depressive symptoms(n=4), anxiety symptoms (n=8), and sleep disturbances (n=2) for the path via depressive symptoms and the path via anxiety symptoms and sleep disturbance, respectively.

[b] β coefficients are the unstandardized coefficients.

The depressive symptoms, anxiety symptoms, and sleep disturbances were continuous variables

All models were adjusted for age, sex (male or female), education level (elementary or lower, junior high, senior high, and college or higher), current smoking (yes or no), current drinking (yes or no), sitting (h/d), walking for more than 10 min at least one day in the last week (yes or no), moderate or vigorous activity (yes or no), marital status (married or widowed, divorced, or unmarried), living status (living alone, living with one roommate, living with two roommates, living with three roommates), overweight (yes or no), number of comorbidities, and hearing impairment (yes or no).

Supplementary Table 5 Sensitive analysis of mediation analysis models of associations between loneliness components and cognitive function, mediated by depressive symptoms, anxiety symptoms, and sleep disturbances [a]

Path models	Personal feelings of isolation		Lack of relational connectedness	
	β coefficients [b] (95% CI)	*P*-value	*β* coefficients [b] (95% CI)	*P*-value
Path via depressive symptoms (n = 224)				
Path α (reference Low)	1.28 (0.27 to 2.29)	**0.01**	1.58 (0.61 to 2.56)	**0.002**
Path β	-0.16 (-0.28 to -0.03)	**0.01**	-0.17 (-0.29 to-0.04)	**0.01**
Direct effect (reference Low)	-1.00 (-1.92 to -0.07)	**0.04**	-0.54 (-1.46 to 0.38)	0.25
Indirect effect (reference Low)	-0.20 (-0.51 to -0.01)	**Significant**	-0.26 (-0.58 to -0.04)	**Significant**
Total effect (reference Low)	-1.20 (-2.13 to -0.27)	**0.01**	-0.80 (-1.71 to 0.11)	**0.08**
Path via anxious symptoms and sleep disturbances (n = 218)				
Path γ (reference Low)	0.91 (0.40 to 1.41)	**0.001**	0.88 (0.39 to 1.36)	**0.001**
Path ε	0.47 (0.21 to 0.73)	**0.001**	0.49 (0.23 to 0.75)	**<0.001**
Path ζ	-0.17 (-0.31 to -0.03)	**0.02**	-0.18 (-0.32 to -0.04)	**0.01**
Direct effect (reference Low)	-0.80 (-1.80 to 0.20)	0.12	-0.51 (-1.47 to 0.46)	0.30
Indirect effect (reference Low)	-0.07 (-0.17 to -0.01)	**Significant**	-0.08 (-0.19 to -0.01)	**Significant**
Total effect (reference Low)	-1.15 (-2.12 to -0.18)	**0.02**	-0.82 (-1.77 to 0.13)	**0.09**

Abbreviations: CI: confidence interval

[a] We did the sensitivity analysis by excluding the outliers (>3 SD or <-3 SD) of depressive symptoms(n=4), anxiety symptoms (n=8), and sleep disturbances (n=2) for the path via depressive symptoms and the path via anxiety symptoms and sleep disturbance, respectively.

[b] *β* coefficients are the unstandardized coefficients.

The depressive symptoms, anxiety symptoms, and sleep disturbances were continuous variables

All models were adjusted for age, sex (male or female), education level (elementary or lower, junior high, senior high, and college

or higher), current smoking (yes or no), current drinking (yes or no), sitting (h/d), walking for more than 10 min at least one day in the last week (yes or no), moderate or vigorous activity (yes or no), marital status (married or widowed, divorced, or unmarried), living status (living alone, living with one roommate, living with two roommates, living with three roommates), overweight (yes or no), number of comorbidities, and hearing impairment (yes or no).

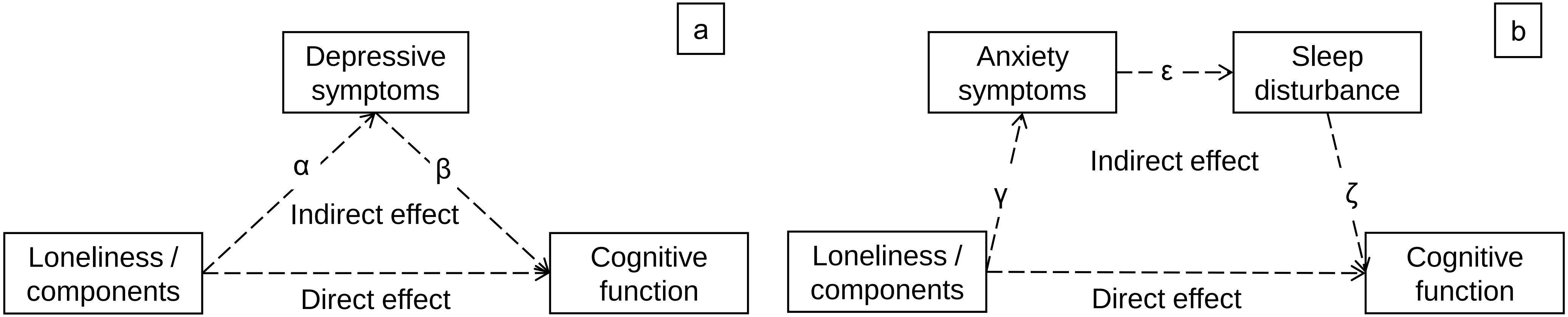

Supplementary Figure 1. Mediation models of depressive symptoms (a), anxiety symptoms, and sleep disturbances (b) between the associations of loneliness / components and cognitive function

The models were adjusted for covariates including age, sex (male or female), education level (elementary or lower, junior high, senior high, college or higher), current smoking (yes or no), current drinking (yes or no), sitting (h/d), walking for more than 10 min at least one day in the last week (yes or no), moderate or vigorous activity (yes or no), marital status (married or widowed, divorced, or unmarried), living status (living alone, living with one roommate, living with two roommates, living with three roommates), overweight (yes or no), number of comorbidities, and hearing impairments (yes or no). The direct effects is the response of cognitive function to changes in loneliness/components while controlling for mediating variables in the model; the indirect effects is the response of cognitive function to changes in loneliness through mediators; and the total effects is the response of cognitive function to changes in loneliness in the presence of mediating variables in the model and is equal to the value of direct effect plus the indirect effect.
